# Advancing Symptom Alleviation with Palliative Treatment (ADAPT) trial to improve quality of life: a study protocol for a randomized clinical trial

**DOI:** 10.1186/s13063-019-3417-1

**Published:** 2019-06-13

**Authors:** Bridget A. Graney, David H. Au, Anna E. Barón, Andrew Cheng, Sara A. Combs, Thomas J. Glorioso, Grady Paden, Elizabeth C. Parsons, Borsika A. Rabin, Debra P. Ritzwoller, Jessica-Jean Stonecipher, Carolyn Turvey, Carolyn H. Welsh, David B. Bekelman

**Affiliations:** 1 Division of Pulmonary Sciences and Critical Care Medicine, Department of Medicine, University of Colorado School of Medicine, Anschutz Medical Campus, Aurora, CO USA; 2 Department of Veterans Affairs, Puget Sound Health Care System, Seattle, WA USA; 3 Division of General Internal Medicine, Department of Medicine, University of Colorado School of Medicine, Anschutz Medical Campus, Aurora, CO USA; 4 Department of Biostatistics and Informatics, Colorado School of Public Health, University of Colorado Anschutz Medical Campus, Aurora, CO USA; 5 Division of Cardiology, Department of Medicine, University of Washington School of Medicine, Seattle, WA USA; 6 Division of Renal Diseases and Hypertension, Department of Medicine, University of Colorado School of Medicine, Anschutz Medical Campus, Aurora, CO USA; 7 Division of General Internal Medicine, Department of Medicine, University of Washington School of Medicine, Seattle, WA USA; 8 Division of Pulmonary, Critical Care and Sleep Medicine, University of Washington, Seattle, WA USA; 9 Department of Psychiatry, University of Iowa Carver College of Medicine, Iowa City, IA USA; 10 Center for Access and Delivery Research and Evaluation, Department of Veterans Affairs, Iowa City Health Care System, Iowa City, IA USA; 11 Department of Family Medicine and Public Health, University of California San Diego School of Medicine, San Diego, CA USA; 12 Institute for Health Research, Kaiser Permanente Colorado, Denver, CO USA; 13 University Writing Program, University of Florida, Gainesville, FL USA; 14 Department of Veterans Affairs, Eastern Colorado Health Care System, Aurora, CO USA; 15 University of Colorado Anschutz Medical Campus, Aurora, USA; 16 National Jewish Health, Anschutz Medical Campus, 12700 East 19th Avenue, Research 2, 9th Floor, Box C272, Aurora, CO 80045 USA

**Keywords:** Heart failure, chronic obstructive pulmonary disease, interstitial lung disease, palliative care, quality of life

## Abstract

**Background:**

People living with chronic heart failure (CHF), chronic obstructive pulmonary disease (COPD), and interstitial lung disease (ILD) suffer impaired quality of life due to burdensome symptoms and depression. The Advancing Symptom Alleviation with Palliative Treatment (ADAPT) trial aims to determine the effect of a multidisciplinary, team-based intervention on quality of life in people with these common diseases.

**Methods/design:**

The ADAPT trial is a two-site, patient-level randomized clinical trial that examines the effectiveness of the ADAPT intervention compared to usual care on patient-reported quality of life at 6 months in veterans with CHF, COPD or ILD with poor quality of life and increased risk for hospitalization or death. The ADAPT intervention involves a multidisciplinary team—a registered nurse, social worker, palliative care specialist, and primary care provider (with access to a pulmonologist and cardiologist)—who meet weekly to make recommendations and write orders for consideration by participants’ individual primary care providers. The nurse and social worker interact with participants over six visits to identify and manage a primary bothersome symptom and complete a structured psychosocial intervention and advance care planning. The primary outcome is change in patient-reported quality of life at 6 months as measured by the Functional Assessment of Chronic Illness Therapy-General questionnaire. Secondary outcomes at 6 months include change in symptom distress, depression, anxiety, disease-specific quality of life hospitalizations, and advance care planning communication and documentation. Intervention implementation will be assessed using a mixed-methods approach including a qualitative assessment of participants’ and intervention personnel experiences and a quantitative assessment of care delivery, resources, and cost.

**Discussion:**

The ADAPT trial studies an innovative intervention designed to improve quality of life for veterans with common, burdensome illnesses by targeting key underlying factors—symptoms and depression—that impair quality of life but persist despite disease-specific therapies. Leveraging the skills of affiliate health providers with physician supervision will extend the reach of palliative care and improve quality of life for those with advanced disease within routine outpatient care. The hybrid effectiveness/implementation design of the ADAPT trial will shorten the time to broader dissemination if effective and create avenues for future research.

**Trial registration:**

ClinicalTrials.gov, NCT02713347. Registered March 19, 2016.

**Electronic supplementary material:**

The online version of this article (10.1186/s13063-019-3417-1) contains supplementary material, which is available to authorized users.

## Background

Despite advances in disease-specific therapies, patients with chronic heart failure (CHF), chronic obstructive pulmonary disease (COPD), and interstitial lung disease (ILD) experience significant symptom burden that contributes to impaired quality of life [1–6]. While these diseases have different pathophysiologic mechanisms, patients experience similar symptoms, including dyspnea at rest and with exertion [7, 8], fatigue [9], pain [10, 11], sleep disturbances, and reduced functional status [12]. Dyspnea and fatigue in particular are pervasive in each of these diseases and are key factors leading to reduced quality of life for patients.

In addition to these symptoms, patients with CHF, COPD, and ILD frequently experience depressive symptoms and many meet clinical criteria for depressive disorders [13–16]. The presence of depressive symptoms independently alters a person’s experience and interpretation of symptoms, worsening the severity of each [12, 13, 17]. Furthermore, studies suggest that increasing underlying disease severity is associated with increased severity of depression [15, 18, 19]. This results in ever-increasing burden of both disease and symptomatic impairment: as disease progresses, symptoms and depression worsen, further reducing quality of life.

Although the need for disease-specific, symptom-targeted therapies and interventions to improve quality of life and other patient-reported outcome measures is increasingly recognized, few are both efficacious and durable. In lung cancer, a disease with high mortality and significant symptom burden, early palliative care improves quality of life and is associated with a mortality benefit [20, 21]. Due to similarities in bothersome and intrusive symptoms, palliative care may be an effective intervention to improve quality of life in patients with CHF, COPD, and ILD but has not been adequately studied, particularly in the outpatient setting. Currently, palliative care in the inpatient setting, where it is utilized more frequently but when patients are very near the end of life, improves quality of life and decreases costs [22]. Incorporating palliative care management strategies into routine outpatient care may extend the reach and efficacy of palliative care interventions, leading to more sustained improvements in quality of life.

The goal of this randomized clinical trial is to improve quality of life for veterans with CHF, COPD, and ILD through an innovative, multidisciplinary, team-based approach combining disease-specific care for symptoms with palliative symptom management and a psychosocial intervention to treat depression. The ADAPT (Advancing Symptom Alleviation with Palliative Treatment) study aims to evaluate intervention effectiveness and implementation. Understanding implementation (i.e., intervention costs, barriers, and facilitators) will inform necessary changes, identify the potential for dissemination, and advance future research focused on improving quality of life.

## Methods/design

### Conceptual foundation

**Fig. 1:**
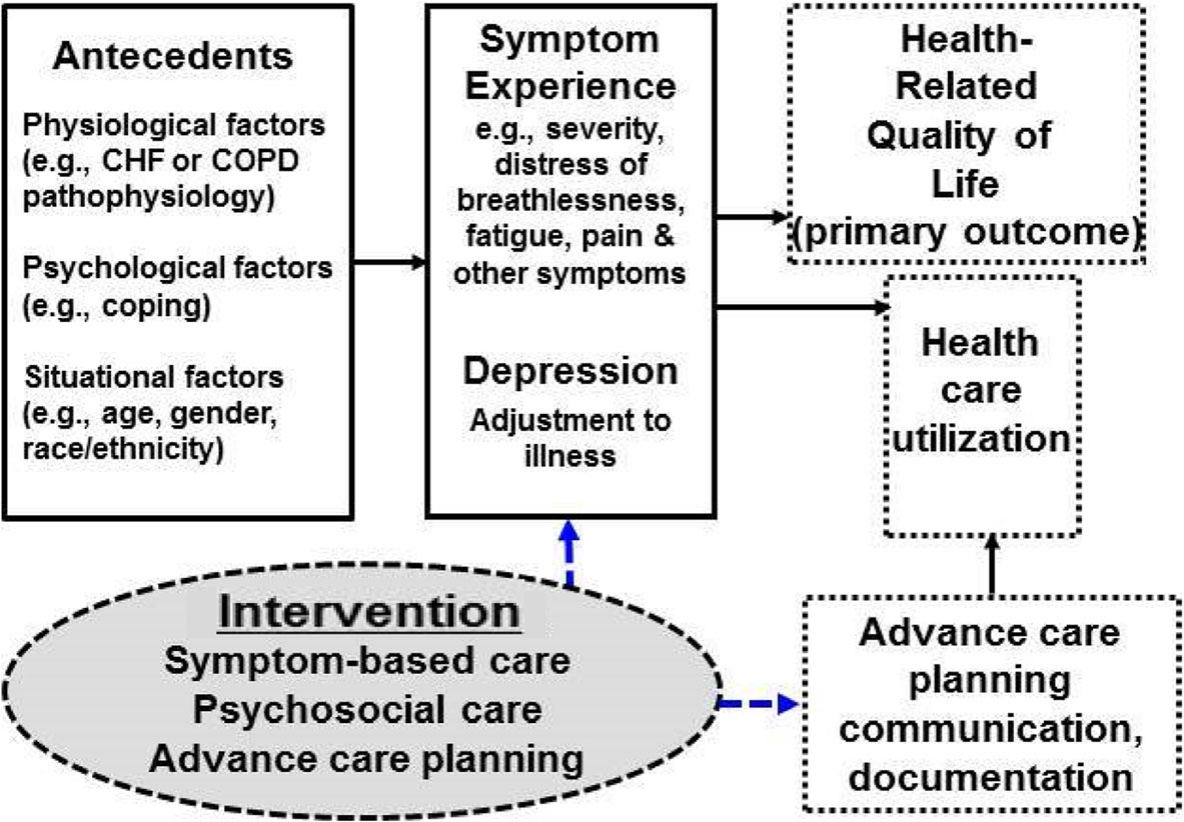
Conceptual model for the ADAPT trial and proposed intervention effects

The ADAPT study utilizes the conceptual framework depicted in Fig. 1 which is based on integrating elements of Lenz’s unpleasant symptom theory [23] into an adaptation of the Wilson and Cleary model of health-related quality of life [24]. Given the relationship between underlying disease severity, symptoms, depression, and impaired quality of life, interventions aimed at improving quality of life must target symptoms and depression concurrently. Therefore, in the ADAPT trial, disease-specific care is provided in conjunction with symptom-focused care, a psychosocial intervention, and advance care planning and communication by employing a multidisciplinary, team-based approach. By addressing individual facets of advanced chronic disease, including symptoms and depression, we hypothesize improvement in participants’ quality of life.

In addition to evaluating effectiveness, the ADAPT study design incorporates a mixed “hybrid effectiveness-implementation” approach [25], evaluating individual intervention components simultaneously with the resources required to implement, maintain, and disseminate the intervention.

### Aims and primary hypotheses

The primary hypothesis is that veterans with CHF, COPD, and ILD randomized to the ADAPT intervention will have improved quality of life at 6 months compared to the control arm of usual care. Secondary hypotheses are that veterans randomized to the intervention will have decreased disease-specific symptom burden, improvement in depression symptoms, increased advance care planning and communication, and decreased hospitalizations compared to usual care.

Based on the primary hypothesis, the first aim of the study is to determine the effectiveness of the ADAPT intervention on improving quality of life. As the ADAPT intervention targets key contributors to impaired quality of life—symptoms and depression—evaluating these and other secondary outcomes of the study are both clinically meaningful and important to patients, informal (e.g., family) caregivers, and providers.

The secondary aim of the study is to examine implementation of the ADAPT intervention. Utilizing a mixed-methods approach, key stakeholders, including participants, informal caregivers, study personnel, and providers, will provide feedback on the intervention components to assess facilitators and barriers to implementation. Additionally, given that a primary barrier to implementation is the costs and resources required for start-up and maintenance of an intervention [26], these will be evaluated. Incorporating these two facets of intervention testing—effectiveness and implementation testing—can reduce the time delay from innovation discovery to implementation, advance scientific knowledge, and increase policy relevance of clinical research.

### Setting

The study is a randomized clinical trial conducted at two Veterans Health Administration (VA) facilities, the VA Eastern Colorado Health Care System (VA ECHCS) and VA Puget Sound. Each health care system encompasses a tertiary care medical center and seven community-based outpatient clinics.

### Study overview

**Fig. 2:**
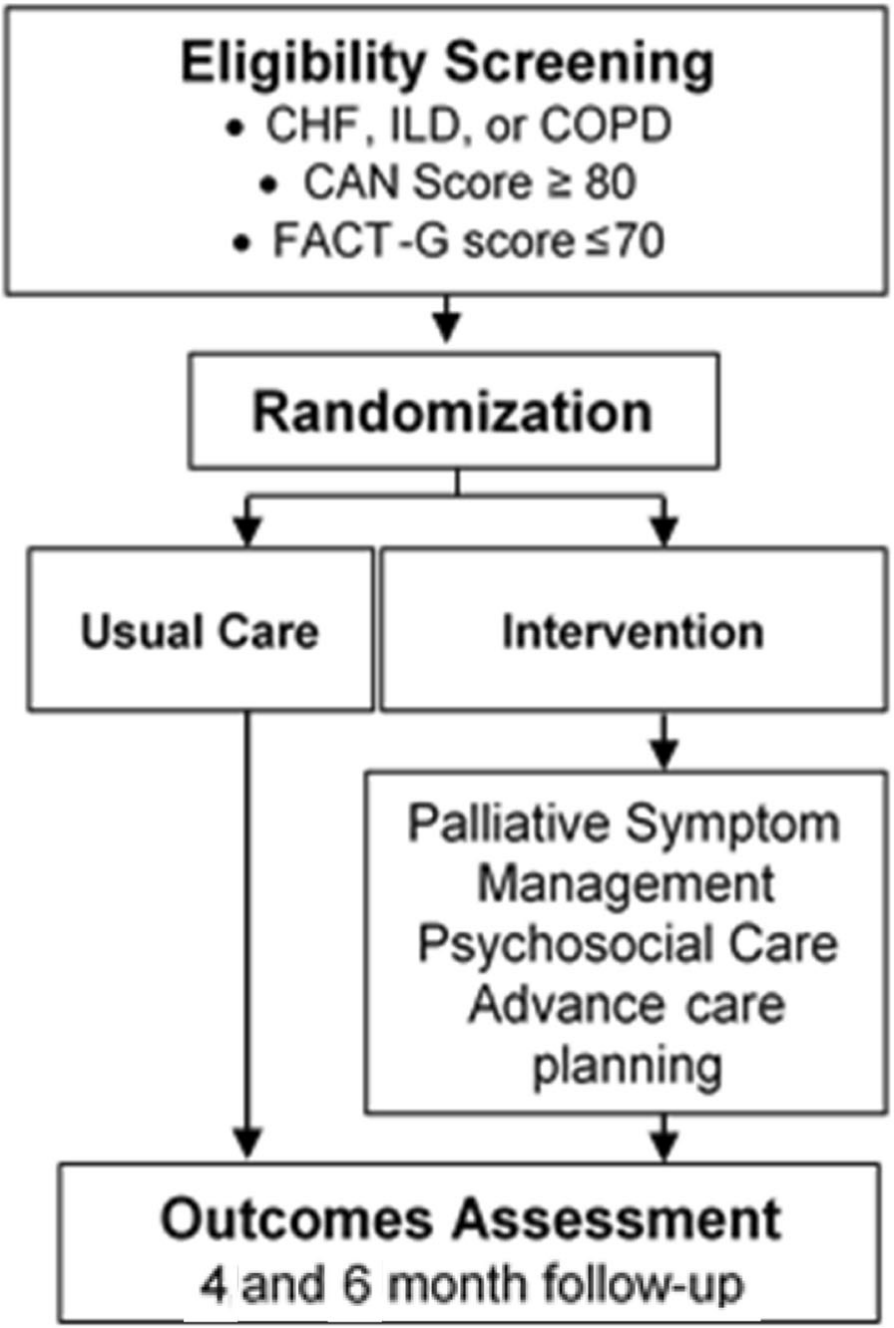
Flowchart of the ADAPT study

Veterans with CHF, COPD, or ILD with impaired quality of life who are at increased risk for hospitalization or death are eligible for participation in the study. Enrolled subjects are randomized 1:1 to usual care or the intervention arm (team-based care plus usual care; Fig. 2). Randomization occurs at the patient level with computer generated random block sizes, stratified by study site and disease.

### Participants

The study will enroll 300 veterans with CHF, COPD, or ILD with self-reported poor quality of life and increased risk of hospitalization or death who are able to participate in the intervention. Complete inclusion and exclusion criteria are provided in Table 1.

**Table 1:** Eligibility criteria

Inclusion criteria	Definition
Diagnosis of CHF or COPD within 2 years prior to enrollment Diagnosis of ILD within 2 years prior to enrollment	Hospitalization discharge diagnosis or ≥ 2 outpatient visits^a,b^ Hospitalization discharge diagnosis or ≥ 1 outpatient diagnosis by a pulmonologist^c^
Among those with CHF or COPD, high risk for hospitalization and death	CAN score ≥ 80
Poor quality of life	FACT-G score ≤ 70
Symptomatic	Bothered by at least one of the target symptoms: pain, fatigue, depression, shortness of breath, trouble sleeping
Primary care or other provider who is willing to facilitate intervention medical recommendations	PCP listed in Electronic medical record review or self-report
Able to read and understand English	Self-report
Consistent access to and able to use a standard telephone	Self-report
Exclusion criteria	
Previous diagnosis of dementia	Inpatient or outpatient diagnostic code^d^
Active substance abuse	Electronic medical record review for substance abuse in the previous 6 months
Comorbid metastatic cancer	Electronic medical record review
Diagnosis of obesity hypoventilation syndrome	Inpatient or outpatient diagnostic code^e^
Nursing home resident	Electronic medical record review or self-report
Heart or lung transplant or LVAD	Electronic medical record review or self-report
Participation in the intervention arm of the CASA trial^f^	Electronic medical record review
Enrolled in palliative care, hospice, or home-based primary care	Electronic medical record review or self-report
Prisoner	Electronic medical record
Pregnant	Electronic medical record or self-report

Footnote: ^a^ICD-9 codes for CHF (428.XX) and corresponding ICD-10 codes; ^b^ICD-9 codes for COPD (491.XX, 492.XX, 493.2, 496.XX) and corresponding ICD-10 codes; ^c^ICD-9 codes for ILD (515, 516.30, 516.31, 516.32, 516.34, 516.37) and the corresponding ICD-10 codes; ^d^ICD-9 codes for dementia (290.0–290.43, 291.2, 046.1, 294.0, 294.1x, 294.2x, 294.8, 331.0, 331.1x, 331.2, 331.6, 331.7, 331.82, 331.89, 331.9) and the corresponding ICD-10 codes; ^e^ICD9 278.03, ICD10 E66.2, or BMI ≥ 45 and diagnostic codes for COPD; ^f^Clinicaltrials.gov, NCT01739686. *CAN* care assessment need, *CHF* congestive heart failure, *CASA* collaborative care to alleviate symptoms and adjust to illness, *COPD* chronic obstructive pulmonary disease, *FACT-G* Functional Assessment of Cancer Therapy—General, *ILD* interstitial lung disease, *PCP* primary care provider

To identify veterans with increased risk for hospitalizations or death, we use a VA-developed prognostic tool, the Care Assessment Need (CAN) score [27]. The CAN creates a probability estimate of hospital admission or death within one year. Patients with CHF or COPD are potentially eligible to participate in this study if they are in the top 20th percentile of risk for hospitalization or death in the next year. As the CAN score is not validated for use in ILD patients, it will not be used as an eligibility criterion for those with ILD. However, patients with ILD, particularly fibrotic ILD, have high rates of hospitalization [28, 29] with significant associated in-hospital or subsequent mortality and are therefore eligible for participation. ILD was approved for addition to the study protocol on May 25, 2018 to expand eligibility criterion.

After meeting diagnostic and at-risk criteria, potential subjects are screened for quality of life using the Functional Assessment of Chronic Illness Therapy-General (FACT-G) questionnaire [30]. A score ≤ 70 (with lower scores indicating worse quality of life) indicates potential eligibility. This cutoff was chosen because a score ≤ 70 identifies poor quality of life as validated by declining performance status and increasing disease burden [30].

### Recruitment

To facilitate recruitment and assess eligibility, a HIPAA waiver was granted at both study sites to allow screening of administrative databases and review of medical records. Eligible subjects at both study sites are identified electronically using validated combinations of diagnostic codes for CHF, COPD, or ILD [31, 32]. This generates a list of potential subjects who meet eligibility criteria based on available administrative data. Study personnel then screen individual medical records to confirm eligibility.

After confirmation, patients’ primary care providers are contacted to confirm the study team can contact their patients and to explain the study. With primary care provider approval, veterans are mailed letters describing the study and providing contact information for study staff if they are interested in participating. If an eligible veteran does not contact the study team, they are contacted by telephone. We anticipate that it will take 25 months to recruit 300 subjects.

### Ethics

This study has been approved by the Colorado Multiple Institutional Review Board protocol #15–1891, the VA Puget Sound Multiple Institutional Review Board protocol #00857 and is registered at ClinicalTrials.gov (NCT02713347). The study is funded by VA HSR&D IIR 14–346 (Bekelman, Principal Investigator).

### Intervention

The intervention is a multidisciplinary, team-based approach to addressing symptoms and psychosocial needs of participants (Table 2). The team-based approach is based on the evidence-based collaborative care model of health care delivery [33, 34]. The intervention personnel include a registered nurse (RN) and Master’s level social worker (MSW). They integrate into a larger collaborative care team (“Team”) that includes a representative primary care provider (PCP) and palliative care specialist. Specialist support with a cardiologist or pulmonologist is available for the Team for additional management recommendations if needed. Each site has a Team that meets weekly for 30–60 min, integrating palliative symptom management with disease-specific care plans.

**Table 2:** Intervention overview

Intervention component	Personnel
Algorithm-guided symptom management: breathlessness, fatigue, pain, trouble sleeping	Registered nurse (RN)
Structured psychosocial care, targeting depression and adjustment to illness; advance care planning	Social worker
Team collaborative care model: 30–60 min weekly team meetings	RN, LCSW, palliative and primary care providers. As needed access with cardiology and pulmonary specialists

The Team is responsible for recording recommendations in a progress note in the electronic medical record, implementing non-pharmacological recommendations, and writing orders for medications or tests that the participants’ individual PCPs review and sign at their discretion. This integration creates improved communication between the intervention Team and the PCP and an additional level of safety for participants.

Based on the conceptual model of the study, the intervention includes three pillars: symptom-based care, psychosocial care and advance care planning. Each facet of the ADAPT intervention takes place in collaboration with the Team and participants’ individual PCPs. RNs and MSWs interact directly with the participants across several visits as subsequently described.

#### Symptom-based care

The intervention personnel make an initial visit either in-person, via phone, or VA telehealth with participants and, if interested, their informal caregivers. Following the initial evaluation with the RN, participants choose a primary symptom—pain, fatigue, mood (including depression or anxiety), shortness of breath, or trouble sleeping—to target for intervention. Participants have the option to change the primary symptom of focus on subsequent visits. After a symptom assessment, the RN follows specific symptom-based algorithms to develop an initial management plan with the Team during a weekly meeting. The RN provides approximately six total visits (two per month) over the study period. For each visit, the RN is responsible for following-up medical orders and other recommendations, assessing changes in symptoms, communicating with participants’ PCPs and other health care providers, and providing education in disease, health care system navigation, and advance care planning to the participants.

#### Psychosocial care

The MSW conducts an initial psychosocial assessment [35] and provides six phone-based counseling sessions. The structured counseling was specifically developed and tested in patients with CHF or COPD to improve depression [36] and empower them to discuss issues related to their illness with their care providers. Antidepressant medication can be recommended to supplement counseling if the Team determines it is an appropriate, evidence-based treatment recommendation. The MSW assists participants in clarifying health care goals and completing written advance directives.

#### Advance care planning

The RN and MSW have a designated visit with participants to discuss care goals and advance care planning. A structured guideline [37] is used to assess participants’ understanding of their disease and guide a discussion around goals, concerns, and fears. Informal caregivers if present are invited to participate. The conversation(s) are documented in the electronic medical record and participants will be helped to complete advance directives and state-authorized out-of-hospital order forms if appropriate.

### Comparator

Participants randomized to the control group receive usual care at the discretion of their care providers. They participate in the same number of study visits in addition to completing questionnaires and self-assessments at the same intervals as those in the intervention arm. Usual care will be enhanced by providing participant PCPs with the results of baseline depression surveys if a participant screens positive for depression. Individual providers will then assume responsibility for follow-up of the positive depression screen. Participants in the control arm do not have any limitations on care recommendations or referrals, which may include management by subspecialists, mental health providers, or palliative care at the discretion of their PCPs.

### Outcome measures

The outcome measures and frequency of data collection are summarized in Table 3. Patients self-complete the survey measures and mail surveys to study staff. Surveys are labeled with a study identification number and double-entered by study staff unaware of participant’s treatment arm assignment.

**Table 3:**
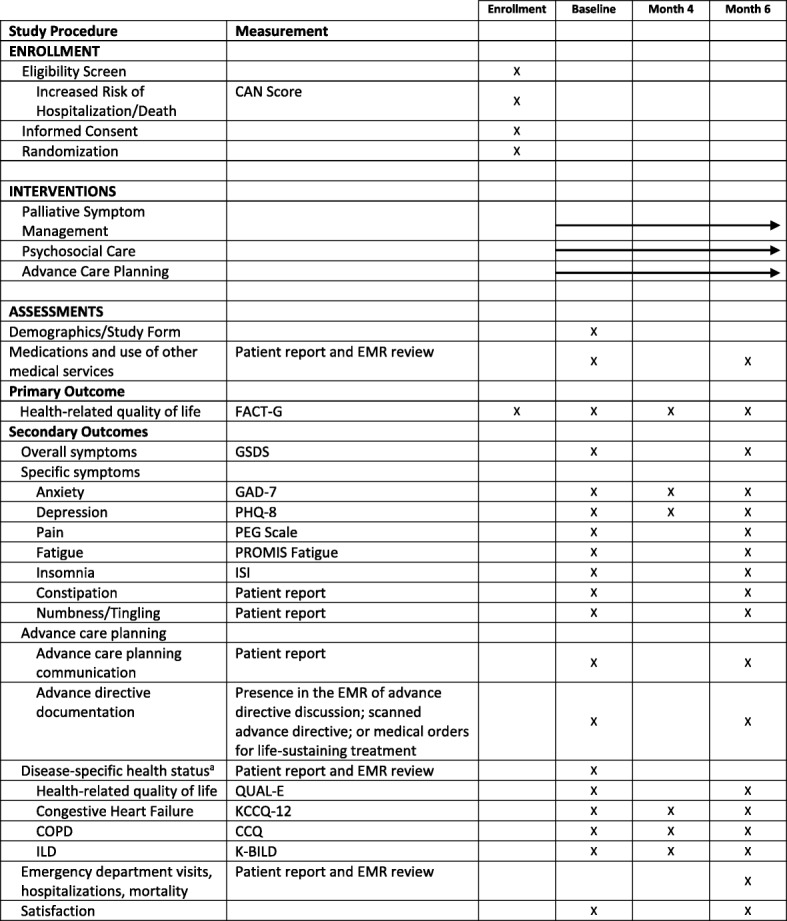
Schedule of enrollment, interventions, and assessments

Adapted from SPIRIT (Standard Protocol Items Recommendations for Interventional Trials) figure. SPIRIT checklist included in Additional file 1.

^a^Participants will complete disease-specific questionnaires based on primary diagnosis. *CAN* care assessment need, *CCQ* Clinical COPD Questionnaire, *COPD* chronic obstructive pulmonary disease, *EMR* electronic medical record, *FACT-G* Function Assessment of Cancer Therapy—General, *GAD-7* Generalized Anxiety Disorder-7, *GSDS* General Symptom Distress Scale, *ILD* interstitial lung disease, *ISI* Insomnia Severity Index, *K-BILD* King’s Brief Interstitial Lung Disease, *KCCQ-12* Kansas City Cardiomyopathy Questionnaire, *PEG* a three-item scale assessing pain intensity and interference, *PHQ-8* Patient Health Questionnaire-8, *PROMIS Fatigue* patient-reported outcomes measurement information system fatigue scale, *QUAL-E* Quality of Life at the End of Life

#### Primary outcome

The primary outcome is patient-reported quality of life. Quality of life is assessed using the Functional Assessment for Chronic illness Therapy-General (FACT-G) questionnaire. The FACT-G is a reliable quality of life questionnaire [38] that is valid in CHF and COPD [39]. It is responsive to specialist palliative care interventions [40] and is correlated with disease severity. Population norms have been established, allowing it to be used across study populations [38]. It measures four subscales that contribute to quality of life: physical, social/family, emotional, and functional well-being. The questionnaire consists of 27 self-report items, each scored on a five-point Likert scale. Individual subscale scores and a total FACT-G score can be determined. Total FACT-G scores range from 0 to 108, with higher scores indicating better quality of life.

#### Secondary outcomes

Secondary outcomes were chosen based on domains within the conceptual model of the ADAPT trial.

##### Symptom experience

Symptom distress will be measured using the General Symptom Distress Scale (GSDS). This is a valid and reliable single item measure of overall symptom distress [41]. It asks, “In general, how distressing are all of your symptoms to you?” It is rated on a numeric rating scale from 0 (“not at all distressing”) to 10 (“extremely distressing”). In addition to the overall impact of symptoms, participants will complete questions to assess specific symptoms, including anxiety, fatigue, pain, insomnia, constipation, and numbness/tingling (Table 3).

##### Depression

Depression will be assessed using the Patient Health Questionnaire-8 (PHQ-8) [42]. The PHQ-8 is a valid, reliable instrument that provides a continuous measure of depressive symptoms. It is both sensitive and specific for a diagnosis of major depressive disorder.

##### Health-related quality of life

The FACT-G is the primary outcome assessment for the ADAPT trial. Participants will also complete subscales of the Quality of Life at the End of Life (QUAL-E) questionnaire, a measure of patient-reported quality of life in advanced illness [43, 44]. It is a valid and reliable instrument utilizing four domains, each scored separately. To assess disease-specific quality of life, study participants will complete one of the following self-report instruments based on their respective underlying diagnosis: the Kansas City Cardiomyopathy Questionnaire (KCCQ-12); the Clinical COPD Questionnaire (CCQ); or the King’s Brief Interstitial Lung Disease (K-BILD).

The KCCQ-12 is a valid instrument that measures CHF-specific health status [45]. It is a shortened version of the KCCQ-23, which is a valid, reliable instrument [46, 47] that is sensitive to clinical change and predicts hospitalization and mortality. The CCQ is a ten-item self-report instrument that is a valid, reliable, and responsive [48, 49] measure of COPD symptoms, functioning, and emotional well-being. The K-BILD is a valid instrument used to assess health status and quality of life for patients with ILD [50].

##### Hospitalizations

Patient self-report of hospitalizations will be used as a measure of health care utilization. Medical records from VA and non-VA facilities will be reviewed with permission to validate the patient report.

##### Advance care planning communication and documentation

Readiness to engage in advance care planning is measured using a valid patient reported survey [51, 52]. Goal concordant care is measured using one question that elicits patient preference for extending life or assuring comfort and another question that assesses patient perception of their current medical care with the same choices [53]. Advance care planning documentation is defined by the presence of a documented advance care planning discussion, scanned advance directive (either a living will or durable power of attorney for health care), or medical orders for life-sustaining treatment within the electronic medical record.

### Evaluation of intervention implementation

Implementation of the intervention will be evaluated using a mixed-methods approach. Using qualitative methods, participants and key study personnel will provide feedback of the implementation process and intervention components utilizing selected Consolidated Framework for Implementation Research (CFIR) domains [54]. Study participants will complete an interview after the 6-month visit with a focus on the value of different intervention personnel, the content and value of the intervention, communication and care coordination, and their perceptions with respect to sustainability of the intervention.

The intervention team will participate in a structured focus group discussion at the completion of the study. The discussion will elicit feedback about what parts of the intervention worked well, might be streamlined, enhanced, or eliminated and views about patient, informal caregiver, and PCP receptivity and responsiveness.

An intervention database will be used throughout the study to track intervention content and processes. PCPs with patients who complete the intervention will be surveyed to assess their satisfaction and experience with the implementation process. The survey can be completed via email, phone, or in-person to improve total response rates.

We will evaluate the necessary resources to implement and maintain the intervention. We track the time and other resources associated with intervention implementation and maintenance and will subsequently assign cost. The main implementation resource is personnel training. Maintenance costs include personnel time to provide the intervention, including phone calls, patient visits, team meetings, and care coordination with PCPs.

### Statistical analysis and sample size

Data from all participants will be included regardless of level of participation using an intent-to-treat approach. The primary outcome is the difference in FACT-G score at 6 months analyzed as a continuous variable. The sample size was determined to detect a clinically significant difference in the primary outcome of mean FACT-G scores between the intervention and control arms. With an analytic sample size of 115 veterans per arm, we will have 85% power to detect a moderate effect size of 0.4 (two-sided test, alpha = 0.05). We plan to enroll 300 veterans and anticipate 5% will die and 15–20% will have missing outcome data. The minimal clinically important difference on the FACT-G score is 4–6 points [55], and with a standard deviation of 15, a Cohen’s d effect size of 0.4 will be on the high end of clinical significance.

Because of the anticipated high correlation of baseline FACT-G with follow-up FACT-G (r > 0.5), we will include the baseline FACT-G as a precision variable in a linear mixed model of the longitudinal outcome measures. To describe the treatment by disease interaction, we will estimate the treatment effect and its confidence interval within each of the disease groups (CHF, ILD, COPD) using disease-specific health status measures (KCCQ, K-BILD, CCQ) at the 6-month endpoint. In exploratory analyses, we will estimate treatment effect within illness subgroups (CHF, ILD, and COPD) on the primary outcome, and within subgroups of illness, including CHF (preserved vs reduced ejection fraction) and COPD (defined by post-bronchodilator FEV_1_/FVC < 0.70 on spirometry [56]). Missing data will be reviewed to identify potential patterns and examined to assess how these patterns impact our results. Specifically, we will examine plots of group means over time stratified by the time of the last completed observation to determine if biases are evident due to missing data. When data are missing at random, unbiased results can still be obtained from the maximum likelihood method that will be used in the linear mixed model analysis. To account for the possibility of data missing not at random, sensitivity analyses will be performed using pattern mixture models and results will be presented to assess the impact of missing data on the reported conclusions. Among those who were hospitalized or died, we will examine for differences in intensive care utilization and patterns of care at the end of life. We will also examine for intervention effect on longer-term health care utilization (e.g., hospitalization, intensive care utilization).

The quantitative data on intervention component implementation will be examined using descriptive statistics. For example, for team meetings, the number and type of medical orders written and completed will be summarized. For social worker visits, the median, range, interquartile range, and types of modules completed will be displayed. This type of analysis will show what components of the intervention were actually done. It will characterize the true “dose” and content of the intervention that was provided. This will contribute information about what may have led to intervention success or failure.

The qualitative data on intervention components and processes will be analyzed using a combination of inductive and deductive methods. We will create an evolving set of codes linked to units of text (fragments, sentences, or paragraphs). A qualitative analyst and the research assistant will serve as primary coders for qualitative data, and the PI will review coding and codebooks as they are developed. We will follow a systematic process to enhance coder agreement in assigning codes and a peer debriefing process that requires regular meetings with a qualitative analyst, the PI, and the research assistant to review and refine codes, code definitions, and conceptual boundaries for our analysis. The iterative analysis will begin by using a priori codes based on the CFIR model, supplemented by codes reflecting intervention content and structure and questions used for data collection. Codes will be refined and new codes added as new insights emerge. Through systematic coding we will quickly develop working themes and hypotheses about critical intervention components and processes that will be examined (and inform any minor changes in data collection interview guides/survey). These themes will also describe facilitators and barriers to intervention implementation. We will both audio-record and take detailed notes during all data analysis meetings in order to document proposed codes and code revisions, proposed themes and their descriptions, and other decisions made during these working meetings.

We will use several recommended strategies to enhance the validity and credibility of qualitative findings: 1) structured interview guides administered by well-trained interviewers; 2) coding templates and detailed descriptions of codes, coding decisions, and analysis strategies to document all phases of the data analysis (audit trail); and 3) team approaches (at least two analysts) to develop coding templates and independently code subsets of transcripts/notes to determine their agreement and application of codes and code definitions.

In addition to analyzing and summarizing quantitative (implementation tracking database and provider surveys) and qualitative (patient interviews and intervention team focus groups) findings separately, we will also merge findings to draw overarching lessons learned from multiple methods used. In this process, findings will be summarized in a table placing qualitative themes side by side with the quantitative findings to show the extent to which the data converges. Merging these findings will provide “triangulation”: findings from each data source will be used to validate and confirm findings from the other data sources. With the combination of qualitative and quantitative data, we expect to provide a more complete explanation of why certain intervention components and processes are more critical than others, as well as facilitators and barriers to the implementation of the intervention.

We will determine resources and cost of the intervention by first calculating the resources (personnel hours or FTE, and other costs) to implement and maintain the intervention during the study. Personnel costs associated with the program will be calculated based on actual VA nurse (and other staff) wage and benefit rates. Total intervention costs and costs per intervention participant at each site will be calculated. Actual salaries and benefits will be used when calculating personnel costs. Second, we will estimate the resources and costs to implement and maintain the program in a variety of VA settings.

Several sensitivity analyses will estimate the range of intervention costs using alternative assumptions for costs that may vary in different implementation contexts. Three sensitivity analyses are planned: 1) variable labor costs with more or less experienced nurses or physicians; 2) variable efficiency of the nurse and social worker in part-time vs full-time intervention roles—this will be done using actual data that reflect the range of time spent per patient early in the study vs later in the study; and 3) variable patient case mix using the range of time spent per patient. In exploratory analyses, we will estimate which patient-level predictors (e.g., cardiac ejection fraction, spirometry, quality of life) are associated with time spent per patient (outcome) using linear regression modeling.

## Discussion

Improving quality of life for patients with chronic disease is an active area of research but few interventions have been found to be effective or scalable. By using a multifaceted approach to target key areas contributing to impaired quality of life, we hope to improve the lives of veterans living with three common chronic diseases, CHF, COPD, and ILD. By using a team-based approach that leverages the successful collaborative care model and the skills of nurses and social workers collaborating with primary care, palliative care, and other specialists, we hope to increase the reach of palliative care. The ADAPT design attempts to integrate symptom-based palliative care interventions into routine outpatient clinical care. If shown to be efficacious, this model could extend the reach of palliative care from primarily inpatient-based care to a routine aspect of primary care for patients with chronic illness with resultant improvements in quality of life.

By utilizing a randomized clinical trial design and examining aspects of implementation, the ADAPT trial will evaluate effectiveness in addition to process and content of the intervention. If the intervention is shown to improve quality of life, having an understanding of the barriers and facilitators of implementation will allow refinement of the ADAPT design for dissemination and integration into other care models. If the intervention is not successful at improving the primary outcome of quality of life, assessment of the intervention will yield valuable information for how to improve the process or content of the implementation for future studies.

## Trial status

The ADAPT study is registered on ClinicalTrials.gov (NCT02713347), registered March 19, 2016. Recruitment began on September 1, 2016 and will be completed on approximately June 30, 2019. Recruitment is ongoing.

## Additional file

Additional file 1: SPIRIT 2013 checklist: Recommended items to address in a clinical trial protocol and related documents*. (DOC 122 kb)

## Data Availability

Not applicable.

## Abbreviations

ADAPT: Advancing Symptom Alleviation with Palliative Treatment
CAN: Care Assessment Need
CCQ: Clinical COPD Questionnaire
CHF: Congestive heart failure
COPD: Chronic obstructive pulmonary disease
FACT-G: Functional Assessment of Cancer Therapy—General
GSDS: General Symptom Distress Scale
ILD: Interstitial lung disease
K-BILD: King’s Brief Interstitial Lung Disease
KCCQ-12: Kansas City Cardiomyopathy Questionnaire
PCP: Primary care provider
PHQ-8: Patient Health Questionnaire-8
PRO: Patient-reported outcome
QUAL-E: Quality of Life at the End of Life
VA ECHCS: Veteran’s Affairs Eastern Colorado Health Care System
